# The Role of Microglia in Perioperative Neuroinflammation and Neurocognitive Disorders

**DOI:** 10.3389/fnagi.2021.671499

**Published:** 2021-05-28

**Authors:** Sarah Saxena, Veronique Kruys, Joseph Vamecq, Mervyn Maze

**Affiliations:** ^1^Department of Anesthesia, University Hospital Center (CHU de Charleroi), Charleroi, Belgium; ^2^Department of Anesthesia and Perioperative Care, Center for Cerebrovascular Research, University of California, San Francisco, San Francisco, CA, United States; ^3^Laboratory of Molecular Biology of the Gene, Department of Molecular Biology, ULB Immunology Research Center (UIRC), Free University of Brussels (ULB), Gosselies, Belgium; ^4^Inserm, CHU Lille, Univ Lille, Department of Biochemistry and Molecular Biology, Laboratory of Hormonology, Metabolism-Nutrition and Oncology (HMNO), Center of Biology and Pathology (CBP) Pierre-Marie Degand, CHRU Lille, University of North France, Lille, France

**Keywords:** microglia, cognition, perioperative neurocognitive disorders, surgery, neuroinflammation

## Abstract

The aseptic trauma of peripheral surgery activates a systemic inflammatory response that results in neuro-inflammation; the microglia, the resident immunocompetent cells in the brain, are a key element of the neuroinflammatory response. In most settings microglia perform a surveillance role in the brain detecting and responding to “invaders” to maintain homeostasis. However, microglia have also been implicated in producing harm possibly by changing its phenotype from its beneficial, anti-inflammatory state (termed M2) into an injurious pro-inflammatory state (termed M1); it is likely that there are intermediates states between these polar phenotypes and some consider that a gradient exists with a number of intermediates, rather than a strict dichotomy between M1 and M2. In the pro-inflammatory phenotypes, microglia can disrupt synaptic plasticity such as long- term potentiation that can result in disorders of learning and memory of the type observed in Peri-operative Neurocognitive Disorders. Therefore, investigators have sought strategies to prevent microglia from provoking this adverse event in the perioperative period. In preclinical studies microglia can be depleted by removing trophic factors required for its maintenance; subsequent repopulation with a more beneficial microglial phenotype may result in memory enhancement, improved sensory motor function, as well as suppression of neuroinflammatory and oxidative stress pathways. Another approach consists of preventing microglial activation using the non-specific P38 MAP kinase blockers such as minocycline. Perhaps a more physiologic approach is the use of inhibitors of potassium (K^+^) channels that are required to convert the microglia into an active state. In this context the specific K^+^ channels that are implicated are termed Kv1.3 and KCa3.1 and high selective inhibitors for each have been developed. Data are accumulating demonstrating the utility of these K^+^ channel blockers in preventing Perioperative Neurocognitive Disorders.

## Introduction

The aseptic trauma of surgery initiates an inflammatory cascade leading to neuroinflammation that culminates in microglial activation. While the other major cell populations in the CNS (including neurons, astrocytes and oligodendrocytes) share a neuroepithelial origin, microglia are derived from myeloid progenitors in the primitive yolk sac and populate the brain before the formation of the blood-brain barrier during mid-embryogenesis (Ginhoux et al., 2010). Microglia are the resident macrophages of the central nervous system (CNS) parenchyma and share the same yolk sac origin as other long-lived tissue macrophages (Ginhoux et al., 2010; Perdiguero et al., 2015).

Microglia play a key role in surveilling the local environment (Ferrini and De Koninck, 2013), recognizing and scavenging dead cells and pathogens (Hua and Smith, 2004; Wake et al., 2009), as well as synthesizing/releasing cytokines and chemokines (Bilbo and Schwarz, 2009). To understand further the role of microglia it is worthwhile to explore the origins and functions of this key cell-type.

### Neurodevelopmental Role of Microglia

The non-random formation of brain circuitry is instructed by local neuronal activity. During development, microglia prune synapses and modulate over-active neurons, thereby shaping the brain’s circuitry. The dendritic spines, that are crucial for neural development and circuit plasticity (Zuo et al., 2005; Nishiyama and Yasuda, 2015), can be targeted by microglia during the process of “synaptic stripping” in which these cells phagocytose the synaptic boutons (Kettenmann et al., 2013). In the postnatal period, learning-dependent dendritic spine formation is regulated by microglia (Paolicelli et al., 2011; Parkhurst et al., 2013).

The microglia’s ability to modify synaptic plasticity involves both “find-me” and “eat-me” pathways. For example, microglia in the resting phase within zebrafish larvae respond to the local increase of neuronal activity, possibly through ATP “find-me” signals. These signals induce microglia to form bulbous endings and wrap themselves around highly active neurons. With the help of neuronal pannexin-1 hemichannels as an eat-me pathway, neuronal activity is reduced (Li et al., 2012).

The spatiotemporal organization of microglia in the white matter of the developing brain contribute to its roles in processes such as axonal guidance, synaptogenesis, and neurodevelopmental apoptosis (Verney et al., 2010).

Maturation of cortical synapses is highly dependent on the chemokine-induced interaction between the neuronally released fractalkine, CX3CL1 and its cognate microglial receptor, CX3CR1 (Hoshiko et al., 2012). Similarly, outgrowth of dopaminergic axons in the forebrain and the laminar positioning of subsets of neocortical interneurons are affected by microglia (Squarzoni et al., 2014).

### Physiological Roles of Microglia

Microglia continue its regulatory role in synaptic plasticity even after the perinatal period, for example in memory storage. During wakefulness, memories are established by synaptic strengthening that is mediated by synaptogenesis; however, there needs to be a mechanism for removing “fleeting memories” and consolidating those that are required. During the “light phase” (other than REM and nREM 3 stages) of sleep, microglia are activated and phagocytose no-longer- required synapses (Ferrer et al., 1990; Neumann et al., 2009; Choudhury et al., 2020).

In addition to sleep there appear to be other initiating mechanisms for microglia-mediated memory loss (Wang C. et al., 2020). In a contextual fear-conditioning paradigm there was a significant decrease in freezing time for the contextual memory between 5 and 35 days after training and was due to a decrease in synaptic connection between hippocampal engram cells (population of neurons that become activated during learning and require reactivation for memory recall). An increase in phagocytosed synaptic proteins (synaptophysin or PSD95) by microglia at day 35 compared to day 5 was noted. The rate of neuronal activity within the hippocampus correlated positively with freezing behavior and increased with dampening of microglial phagocytosis (minocycline) or depletion of microglia (PLX3397). When an engineered virus that expresses CD55, an inhibitor of complement activation, was injected into the brains of these mice there was an increase in freezing behavior (i.e., improvement in memory) at day 35 after contextual fear conditioning in association with higher levels of activation (using *c-fos*) of engram cells and decreased levels of synaptic proteins within microglia (Wang C. et al., 2020). These data suggest that it is the complement activation that initiates the synaptic pruning by microglia that eliminates weak memories resulting in “physiologic forgetting.” In some conditions pathological remembering is a dominant feature and may in the future be amenable to physiologic forgetting treatments that regulate microglial- and complement-mediated synapse elimination.

These different studies suggest that the adult CNS has a highly plastic and dynamic microglial population that can be entirely repopulated following microglial elimination, even in the aged brain (Schafer et al., 2012; Wang C. et al., 2020).

### Pathological Roles of Microglia

While microglia play an important role in synaptic pruning and phagocytosis in the healthy brain, its activation can also lead to harm (Czeh et al., 2011).

Studies have revealed the correlation of microglial activation to the severity of several neurodegenerative disorders such as Alzheimer’s disease (AD) (Arends et al., 2000; Edison et al., 2008), Parkinson’s disease (Banati et al., 1998; Imamura et al., 2003), and amyotrophic lateral sclerosis (Boillee et al., 2006). In these neurodegenerative settings, microglia, which express Toll-like receptors (TLR) 1–9 (Jack et al., 2005), become activated through stimulation of one or more of these receptors which results in the synthesis and release of pro-inflammatory cytokines that can induce neurotoxicity (Miller, 2004; Harry and Kraft, 2008). In particular microglia strongly express the cell surface TLR2 whereas TLR3 is expressed at higher levels intracellularly. TLR3 signaling leads to the secretion of high levels of IL-12, TNF-alpha, IL-6, CXCL-10, and IL-10, and the expression of IFN-beta, whereas TLR2 signaling leads to IL-6 and IL-10 secretion (Jack et al., 2005). In an AD murine model, TLR2/TLR4 are more highly expressed in microglia (Frank et al., 2009). Inactivation of the TLR2 signaling attenuated microglial activity and improved outcome in an AD model (McDonald et al., 2016).

TLR2/TLR4 signaling pathways in microglia increase infarct size in ischemic settings (Lehnardt et al., 2007).

In obese mice, microglial activation, through the CX3CR1 pathway can result in cognitive impairment (Cope et al., 2018). Mice fed with a high fat diet activated hippocampal microglia and impaired hippocampus-dependent memory through a reduction in long-term potentiation (LTP); microglial activation and attenuation of LTP were accompanied by perturbation of spatial relationships between microglial processes and synaptic puncta, though the internalization of synaptosomes (Barrientos et al., 2010).

### Role of Microglia in Perioperative Neurocognitive Disorders (PND)

**FIGURE 1:**
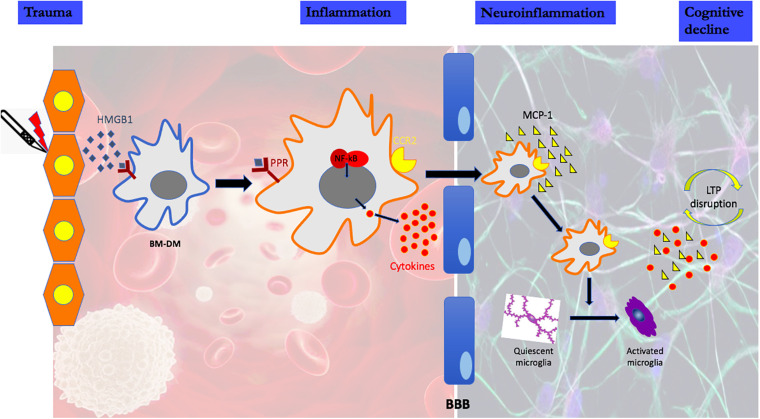
High mobility group protein B1 (HMGB1) is a damage-associated molecular pattern that is immediately released following peripheral trauma. HMGB1 engages the innate immune response by binding to pattern recognition receptors (PRR) on circulating bone marrow-derived monocytes (BM-DMs). Signaling through the PRR results in the disaggregation and intracellular translocation of the transcription factor, nuclear factor k B (NF-kB) which increases the synthesis and release into the circulation of pro-inflammatory cytokines. High cytokine levels disrupt the Blood Brain Barrier (BBB) facilitating the translocation of circulating BM-DMs into brain attracted by the chemokine monocyte chemoattractant protein-1 (MCP-1), which itself is HMGB1-dependent. In the presence of BM-DMs within the hippocampus, quiescent microglia become activated as evidenced by the change in morphology and the release of proinflammatory cytokines. Lon-term potentiation (LTP), a synaptic plasticity mechanism required for creating and storing memory is disrupted by the proinflammatory cytokines in the hippocampus.

Using a rodent model of aseptic trauma, we have investigated the potential role of microglia for cognitive decline assessed as a decrease in freezing behavior to a previously trained aversive stimulus. The putative pathogenic mechanisms responsible for the cognitive decline associated with the acute form of PND are illustrated in Figure 1 (Wan et al., 2007; Cibelli et al., 2010; Terrando et al., 2010, 2011; Degos et al., 2013; Vacas et al., 2014; Feng et al., 2017a,b; Hu et al., 2018a,b; Ieng et al., 2020). It remains an open question whether and how microglia affect later-onset variants of PND as there are, as yet, no preclinical models that consistently produce a phenotype akin to the clinical disorders of Delayed Neurocognitive Recovery and Postoperative Neurocognitive Disorder (see Discussion).

### Microglial Polarization Into M1 vs. M2

Analogous to macrophages, microglial activation has been dichotomized into a “neurotoxic” M1 and a “neuroprotective” M2 phenotype (Tang and Le, 2016); however, this binary outcome has been challenged arguing for a more nuanced spectrum from M1 to M2 with intermediate states that depend on environmental factors (Hanisch and Kettenmann, 2007; Ransohoff, 2016).

Nevertheless, the following are examples in which the phenotypic expression was changed from the M1 to M2 to thwart pathological processes.

1. Hypoxia has been shown to favor the M1 state (Zhang et al., 2017). In BV-2 cells, a microglia-like cell-line, oxidative stress induced the expression of iNOS, led to an increased level of CD68 (an M1 marker) and triggered the Tyr-701-phosphorylation and S-glutathionylation of STAT1. Silencing of STAT1 protein expression counteracted hypoxia-M1 microglia phenotype (Butturini et al., 2019).
2. A similar transformation obtains in a preclinical rat model of cerebral ischemia from middle cerebral artery occlusion, in which the M1 phenotype is induced through a CysLT_2_R-ERK1/2 pathway that results in microglial elaboration of neurotoxic factors such as NO, IL-1β, TNF-α, and IL-12 (Zhao et al., 2020). Treatment with mesenchymal stem cell-derived exosomes suppresses the neurotoxic factors and enhances the synthesis and release of M2 phenotypic markers, such as IL-10, TGF-β, and BDNF, that results in an improved outcome (Zhao et al., 2020). Similarly, in a preclinical mouse model of ischemia-reperfusion injury, following 30-min of middle cerebral artery occlusion, the M1-microglial phenotype was transformed into M2 by curcumin-encapsulated nanoparticles resulting in an improvement in both infarct size and functional deficit (Zhao et al., 2020).
3. In a rat model of intracerebral hemorrhage, there was a rapid transformation of microglia to the M1 phenotype with increased expression of its biomarkers; following administration of JWH133, a cannabinoid 2 receptor agonist, the microglia begin to transcribe markers of the M2 phenotype through activation of the PKA/CREB signaling pathway and result in improvement (Lin et al., 2017).
4. In a mouse model of AD accumulation of the neurotoxic amyloid β (Aβ) oligomers promote M1 glial transformation including elaboration of IL-1b; when signaling through the IL-1 receptor is precluded, transcription levels of M2-polarized macrophage related-inflammatory factors, such as IL-10 and Ym1 increase and are accompanied by improvements in learning and memory (Wang H. et al., 2020).
5. In the autoimmune inflammatory model of encephalomyelitis, inhibition of the purinergic receptor P2 × 4R signaling favored microglia M1 transformation and exacerbated demyelination; conversely, stimulation of P2 × 4R with ivermectin favored microglial polarization to the M2 phenotype, promoted remyelination and improved functional deficits (Zabala et al., 2018).
6. Ketamine has been advocated for treatment-resistant depression (Schoevers et al., 2016). In a mouse model of depression induced by lipopolysaccharide, ketamine suppressed the LPS-induced upregulation of pro-inflammatory M1 markers and changed the configuration of microglia; these changes were associated with an improvement in the behavioral tests of depression (Verdonk et al., 2019).

### Attenuating Microglial Function

As activation of microglia is a pivotal step in the development of postoperative cognitive decline, different strategies have been attempted to attenuate microglial function.

#### Baseline Effects of Microglial Depletion

Colony stimulating factor 1 (CSF-1) regulates proliferation, differentiation and survival of tissue macrophages and, without CSF-1 signaling, microglia will not survive (Patel and Player, 2009). *In vivo* administration of inhibitors of the CSF-1 receptor (CSF-1R), such as PLX 3397, efficiently deplete microglia. LPS-induced microglial proliferation, as evidenced by increased expression of its stereotypic marker, ionized calcium binding adaptor molecule 1 (Iba1), was prevented by prior feeding of the mice with PLX 3397. In the basal state the CSF-1R inhibitor decreased Iba1 staining by 70% (Elmore et al., 2014); confirmation that microglia undergo apoptotic cell death was established by the upregulation of activated caspase 3. The PLX 3397 effect on microglia can be reversed within 3 days although the morphological appearance as well as the number of cells only returns to normal by 14 days through the presence of microglia progenitor cells. Interestingly, depletion of microglia in adult mice by PLX 3397 neither affects the baseline cognitive and motor function nor permeability of the blood brain barrier (Elmore et al., 2014). Therefore, CSF-1R is a druggable target to prevent neuroinflammation and its consequences as evidenced by the following examples.

#### Improvement of Injury-Induced Functional Deficit Following Microglial Depletion

A hippocampal lesion was produced by diphtheria toxin A-chain in adult mice and were subsequently administered PLX3397 for 30 days post-lesioning. Within 7 days of treatment, ∼70% of microglia had been eliminated and brain levels of IL-1β decreased. Behavioral testing showed improved learning on the elevated plus maze and the Morris water maze (MWM) following PLX3397 treatment; however, the probe trial (a test of recall in the MWM) was unaffected by depletion of microglia (Rice et al., 2015).

**FIGURE 2:**
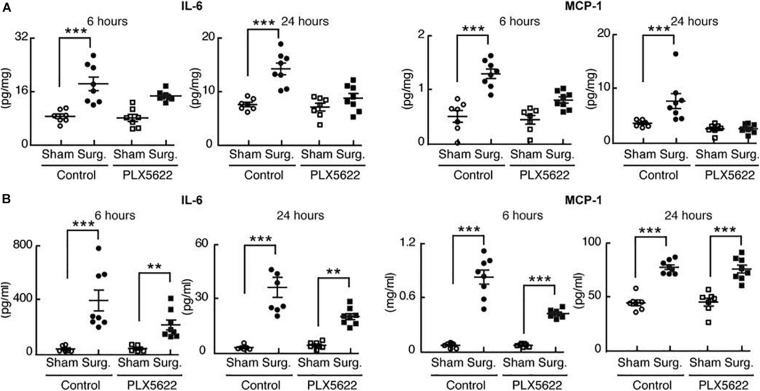
Perioperative microglial depletion abrogates surgically induced hippocampal inflammation. Reproduced with Permission Feng et al. (2017b). **(A)** Tissue ELISA, showing the rise in hippocampal levels of both IL-6 and MCP-1 in response to surgery and the prevention of this postoperative rise by perioperative PLX5622 treatment as analyzed by two-way ANOVA (*P* = 0.006 at 24 h for IL-6, and *P* < 0.01 at both 6 and 24 h for MCP– 1). **(B)** Plasma ELISA, showing the comparative lack of effect of PLX5622 treatment on postoperative plasma IL-6 and MCP-1 levels. Data were analyzed by two-way ANOVA. In all cases, *n* = 7–8/group, and ***P < 0001; **P < 0.001 for surgery vs. corresponding sham-treated control (Feng et al., 2017b).

Using the CSF-1R inhibitor PLX5622, postoperative neuro-inflammation and cognitive decline (POCD) was prevented (Feng et al., 2017b). Mice were fed a standard chow diet containing PLX5622, for 7 days prior to aseptic surgical trauma. Surgery increased hippocampal levels of the proinflammatory cytokines IL-6 and MCP-1 following surgery; these did not occur in the mice pre-treated with PLX5622 indicating that microglial depletion decreases the chemo-attraction for monocyte translocation into the brain and lessens postoperative hippocampal neuroinflammation (Figure 2A).

Interestingly, trauma-induced *peripheral* inflammation was less affected by depletion of microglia with PLX5622 (Figure 2B).

Typically, mice that experience surgical trauma exhibit less “freezing” behavior in a trace-fear conditioning paradigm that elicits memory for an aversive event. Mice treated perioperatively with PLX5622 retained memory for the preoperative aversive event and were no different from non-operative mice.

These data indicate that perioperative microglial depletion can completely prevent the development of this behavioral indicator of Perioperative Neurocognitive Disorders (Feng et al., 2017b).

CSF-1R inhibitors have also been tested in models of neurodegeneration including one that mimicks Alzheimer’s Disease (AD). Fifteen-month-old 3xTg-AD mice, which develop both Aβ plaques and tau tangles (pathological hallmarks of AD), were treated for either 6 weeks or 3 months with PLX5662 or vehicle. At the end of this period, cognitive testing (novel place, novel object recognition, and MWM) and histopathology were performed. In this AD model, mice treated with PLX5622 for 6 weeks or 3 months showed significantly improved place recognition with no difference in novel object recognition. 3xTg-AD mice treated with PLX5622 tended to have faster escape latencies than untreated mice that achieved statistical significance on trials performed on days 6 and 7 on the MWM. Regarding the histopathology, abundant core plaques were present in all mice irrespective of PLX5622 treatment; however, activated microglia were densely packed around plaques in control AD mice, a feature not found in mice treated with PLX5622. Quantification of the number of microglia associated with Aβ plaques revealed a 70% reduction following PLX5622 (Dagher et al., 2015).

As neuroinflammation plays an important role in the development of Parkinson’s Disease (PD), the effects of microglial depletion with PLX3397 have also been studied in a rat model of PD in which the striatum is stereotactically lesioned with 6-hydroxydopamine. PD rats treated with PLX3397 performed better in the adhesive tape removal test and in the forced swim immobility test. Using PET imaging, less neuroinflammation was seen in PD rats treated with PLX3397. Iba-1 staining in the PD group was significantly higher and this was reduced with PLX3397 (Oh et al., 2020).

In mice, microglial depletion suppressed the neuroinflammatory process which resulted in a beneficial effect on the motor and non-motor symptoms of PD (Oh et al., 2020).

In a mouse model of acute intracranial hemorrhage (ICH; either by intracerebral collagenase administration or intracerebral autologous blood administration), prior oral administration of PLX3397 for 21 days reduced the microglial population by ∼90% with no reduction in the monocyte populations in the spleen (Li et al., 2017). In mice with PLX3397-induced depletion of microglia, there was reduced neurological deficits, lesion volume, and perihematomal edema following ICH. Molecular pathways leading to cerebral injury following ICH, including ROS signaling and proinflammatory cytokines in the brain were significantly lower in the ICH mice that were pretreated with PLX3397 (Li et al., 2017).

Four weeks following traumatic brain injury (TBI), produced by controlled cortical injury with a pneumatic impactor, mice were fed the microglial depletor PLX5622 for 7 days. 3 months post-TBI, neuropathological changes were attenuated in the microglial-depleted mice; similarly, there was a decrease in the NOX2- and NLRP3 inflammasome-associated neuroinflammation. Using a variety of complementary neurobehavioral tests, PLX5622-treated TBI mice also had improved long-term motor and cognitive function recovery at 3 months post-injury (Henry et al., 2020).

#### Effect of Repletion With New Microglia on Behavior

Using an alternative approach to deplete microglia, transgenic rats had the diphtheria toxin receptor (*Dtr*) inserted into the promoter region of the fractalkine receptor, *Cx3cr1*, that is expressed on microglia and monocytes. Upon a single application of diphtheria toxin (DT) to these transgenic mice, there was a temporary ablation of both microglia (∼80% reduction) and monocytes (De Luca et al., 2020). Two days following the acute deletion of microglia, behavioral tests [Y maze and novel object recognition test (NOR); object preference], did not reveal any cognitive deficit suggesting that acute microglial ablation does not affect baseline learning and memory. Seven days following acute microglial depletion, at which time amoeboid-appearing microglia had begun to repopulate the hippocampus, performance in the NOR test and spatial memory with novel place recognition was better than in rats that had not received DT suggesting that microglial repopulation after ablation results in better recall. Morphologically, microglial repopulation was associated with an increase in the number of mature neurons in the hilus. There were no differences between the groups in the expression of pre– or post-synaptic markers (*Vglut1*, *Glua2*, *Glun2a*), markers of microglial-neuronal interaction (*Cx3cl1*, *Cx3cr1*), or neurotrophic factors (*Bdnf*, *Ngf*). There was an increase in the hippocampal expression of a microglial recruitment marker, *Cxcl10*, as well as elevated expression of *C1q*, the initiating protein of the canonical complement cascade, and *C3*; these are both known to localize to synapses and mediate the elimination of dendritic spines by phagocytic microglia. Correspondingly, the density of both synaptophysin and PSD-95 in the hilus was significantly reduced with microglial repopulation. While there was a significant increase in arborization of the basal dendrites in the CA3 neurons 7 days following DT, there were no differences in apical or basal dendritic tree length. Microglial repopulation also significantly increased the number of mature bifurcated spines, typically associated with increased spine efficacy, in the CA1 neurons. Astroglia appeared denser in the hilum when microglia repopulate (De Luca et al., 2020).

#### Deterioration of Injury-Induced Functional Deficit and Inflammation Following Microglial Depletion

Microglial depletion is not always associated with improved neurological performance. In a model of PD produced by the neurotoxin 1-methyl-4-phenyl-1,2,3,6-tetrahydropyridine (MPTP), depletion of microglia with PLX3397 exacerbated the impairment of locomotor activities (latency to fall) and the loss of dopaminergic neurons. Microglial depletion augmented the infiltration of CD4^+^ T cells (CD45^*high*^CD3^+^CD4^+^), CD8^+^ T cells (CD45^*high*^CD3^+^CD8^+^), monocytes and macrophages (CD45^*high*^CD11b^+^F4/80^+^), and neutrophils (CD45^*high*^CD11b^+^Ly6G^+^), suggesting that microglia may restrict MPTP-induced leukocyte infiltration. In addition, microglial depletion resulted in a significant increase of activation marker CD69 in CD4^+^ T and CD8^+^ T cells after MPTP treatment. PLX3397 treatment significantly upregulated the expression of proinflammatory cytokines including IL-1β, TNF-α, IL-2, IL-6, IFN-γ, and iNOS in the substantia nigra, suggesting that microglial depletion augments MPTP-induced local inflammation (Yang et al., 2018).

In a stroke model, depletion of microglia resulted in further harm. Following depletion of microglial by a 21-day course of PLX3397, an ischemic-reperfusion injury from transient middle cerebral artery occlusion (MCAO) resulted in enhancement of the neurological deficit and infarct size; these changes were associated with higher ROS, and pro-inflammatory cytokines (IL-1α, IL-1β, IL-6, and TNF-α) and down-regulation of growth factors such as IGF-1 were down-regulated in brain tissues. Astrocytes appeared to be the origin of the increase in neuroinflammation and suggests that the “neuroprotective” effect of microglia may be due to inhibition of astrocyte activation (Jin et al., 2017).

The role of microglia in the setting of a neurotropic viral infection with mouse hepatitis virus (MHV) was explored. In mice that were depleted of microglia with PLX5622, viral replication post-inoculation peaked earlier (day 3 vs. day 5), persisted longer with diminished viral clearance kinetics. Also, microglia-depleted mice had greater morbidity and lethality (Wheeler et al., 2018).

#### Preventing Activation of Microglia

As alluded to earlier, microglia can transform into classical (“M1”) and alternative (“M2”) activated states depending on whether the stimulus is LPS or IL-4, respectively. In these two transformed states there is a differential upregulation of potassium (K) channels on the microglia with the K_*v*_1.3 [sensitive to blockade with phenoxyalkoxypsoralen-1 (PAP-1)] predominating following LPS and the K_*i*__*r*_2.1 (sensitive to blockade with barium) most prevalent following IL-4; the K_*C*__*a*_3.1 channel, activated by Ca^2+^ (sensitive to TRAM-34), was expressed in lesser amounts. As there are selective blockers for each of these K channels it may be possible to attenuate expression and downstream effects of M1 vs. M2 microglia phenotypes (Nguyen et al., 2017).

Intracerebroventricular injection of lipopolysaccharides (ICV-LPS) increased the expression and current density of voltage-gated K_*v*_1.3 channel (but not K_*i*__*r*_2.1) as well as the neuroinflammation as evidenced by Iba-1 immunoreactivity and expression of pro-inflammatory mediators such as IL-1β, TNF-α, IL-6, and iNOS; hippocampal long-term potentiation (hLTP) could not be induced. In mice genetically devoid of K_*v*_1.3, LPS failed to activate microglia, produce neuroinflammation and impair hLTP. Pharmacological intervention using PAP-1, a small molecule that selectively blocks homotetrameric K_*v*_1.3 channels, achieved anti-inflammatory and hLTP-recovery effects similar to that seen in K_*v*_1.3 knockout mice (Di Lucente et al., 2018).

The effect of PAP-1 in preventing microglial activation following transient MCAO in mice (60 min of ischemia followed by reperfusion) was investigated. Enhanced K_*v*_1.3 staining, and higher current density, was observed on activated microglia that were isolated from the ischemic infarcts in mice. PAP-1 dose-dependently reduced infarct area, improved neurological deficit score, and reduced brain levels of IL-1β and IFN-γ without affecting IL-10 and brain-derived nerve growth factor (BDNF) levels or inhibiting ongoing phagocytosis (Chen et al., 2017).

Postoperative cognitive decline requires microglial activation as evidenced by the experiment in which mice undergoing orthopedic surgery were treated with PAP-1 and submitted to trace-fear conditioning (TFC) training and testing.

**FIGURE 3:**
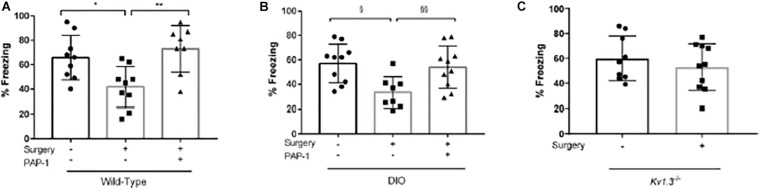
Role of Kv1.3 in postoperative cognitive decline in the trace fear-conditioning (TFC) paradigm. Applied for Permission to Reproduce Ieng et al. (2020). Cohorts of mice were trained in a TFC paradigm immediately before surgery and tested for freezing behavior 3 days after surgery. **(A)** Wild-type mice (12-14 weeks old C57bl/6j) were randomized to three groups (*n* = 8–9 per group) that received no surgery/vehicle, surgery/vehicle, or surgery/phenoxyalkoxypsoralen-1 (PAP-1). **(B)** Wild-type mice with diet-induced obesity (DIO) were randomized to three groups (*n* = 8–10 per group) that received no surgery/vehicle, surgery/vehicle, or surgery/PAP-1. **(C)** Mice deficient in Kv1.3 (Kv1.3–/–) were randomized to two groups (*n* = 910 per group) that received either sham (no surgery) or surgery. After wound closure and every 12 h thereafter, the mice randomized to PAP-1 received 40 mg kg^1^ i.p. vehicle consisted of MIGLYOL in the same volume as PAP-1. On the third day, freezing behavior was tested in the same context as the training. **(A,B)** Analyzed by one-way analysis of variance followed by Bonferroni *post hoc* test. **(C)** Analyzed by unpaired *t*-test. *P1/40.029; **P1/40.005; xP1/40.011; xxP1/40.030 (Ieng et al., 2020).

Freezing behavior of these mice was investigated and mice treated with PAP-1 after undergoing orthopedic surgery had a higher freezing time (*P* = 0.03). This was even more significant in diet-induced obese (DIO) mice (mimicking metabolic syndrome) (Figures 3A,B).

Kv1.3^–/–^ mice also underwent surgery. However, since microglial activation was inhibited in these mice, freezing ratios were similar between “surgical and non-surgical” mice (Figure 3C).

Hippocampal IL-6 levels were investigated in the same murine population.

**FIGURE 4:**
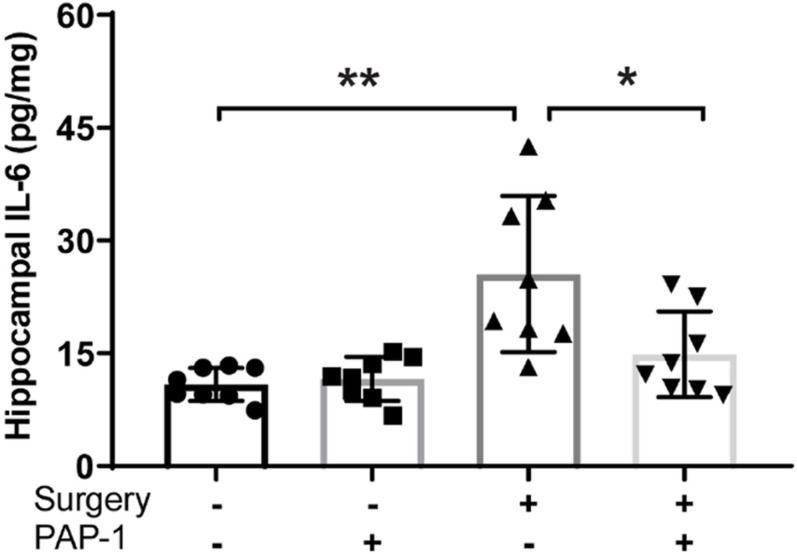
Phenoxyal koxypsoralen-1 (PAP-1) prevents surgery– induced hippocampal inflammation. Applied for Permission to Reproduce Ieng et al. (2020). Mice with diet-induced obesity were randomized to four groups (*n* = 8 per group) that received either no surgery or surgery, and either PAP-1 or vehicle. At 24 h after surgery, the animals were killed and hippocampi were removed and assayed for interleukin-6 (IL-6) by enzyme-linked immunosorbent assay. Data were analyzed by one-way analysis of variance followed by Bonferroni *post hoc* test. *P < 0.001; **P1/40.011 (Ieng et al., 2020).

Surgery causes an increase in hippocampal IL-6 levels, however, pre-treatment with PAP-1 lowered these levels (*P* = 0.011) (Figure 4).

**FIGURE 5:**
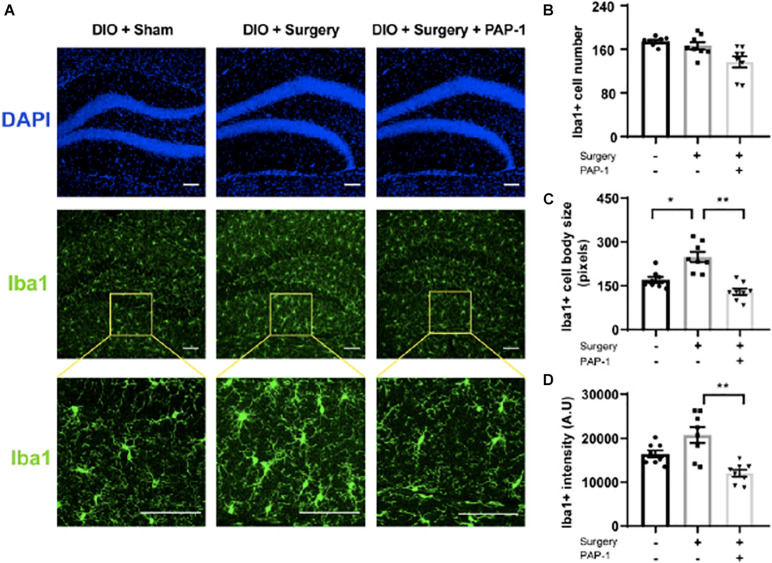
Phenoxyalkoxypsoralen-1 (PAP-1) attenuates surgery-induced microglial activation. Applied for Permission to Reproduce Ieng et al. (2020). Mice with diet-induced obesity (DIO) were randomized to three groups (*n* = 8 per group) that received neither surgery nor PAP-1 (DIO/sham), surgery alone with no PAP-1 (DIO/surgery), or surgery together with PAP-1 (DIO/surgery/PAP-1). After surgery, mice not randomized to receive PAP-1 (DIO/sham; DIO/surgery) were administered MIGLYOL, and mice randomized to PAP-1 received doses intraoperatively and 12 h postoperatively. At 24 h after surgery, mice were killed, and brains were perfused, fixed, and sectioned. Coronal sections (35 mm thick) from the dentate gyrus were stained with anti-Iba1 antibody. Immunofluorescence was performed with Alexa Fluor 488-labeled anti-rabbit antibody. **(A)** Representative photomicrographs from each of the groups. The upper panel represents DAPI staining, the middle panel staining for Iba1, and the lower panel a higher magnification. The internal scale marker represents 50 mm. **(B)** Iba1cells were counted manually by visual inspection of hippocampal sections of anatomically matched photomicrographs, with an average taken from four sequential sections per mouse. **(C)** To analyze microglial cell-body size (in pixels), each section was processed in a systematic way to create a binary image by applying a previously established threshold. **(D)** Iba1 fluorescence intensity [absorbance unit (AU)] was similarly measured with ImageJ from images captured using identical exposure times that also avoided saturating pixel intensities. Data [mean (standard deviation)] were analyzed by one-way analysis of variance followed by Bonferroni *post hoc* test. *P1/40.0012; **P < 0.001 (Ieng et al., 2020).

Iba-1 staining images of the hippocampus of these mice revealed an increase in microglial activity post-operatively, successfully inhibited by PAP-1 treatment (Figure 5).

The same study also reveals interestingly that PAP-1 does not affect peripheral IL-6 levels, confirming the central nervous system activity of PAP-1 (Ieng et al., 2020).

Combined, these data, from the experiments with PAP-1, show that K_*v*_1.3 may be a druggable target for neurological diseases in which microglia-mediated neurotoxicity is implicated in the pathogenesis.

The role of K_*C*__*a*_3.1 has been studied in the setting of transient (60 min) MCAO in mice that was followed by 8 days of reperfusion. Microglia from the infarcted area exhibited higher densities of each of the K^+^ currents than was present in microglia from non-infarcted control brains. Strong K_*v*_1.3 and K_*C*__*a*_3.1 immunoreactivity was also found on activated microglia/macrophages from infarct areas in stroke subjects. Mice treated with 1-[(2-chlorophenyl) diphenylmethyl]-1*H*-pyrazole (TRAM-34), a selective K_*C*__*a*_3.1 blocker, exhibited significantly smaller infarct size, and less of both neuroinflammation as well as neurological deficit after ischemic-reperfusion injury; similar data were found in *K_*C*__*a*_3.1*^–^*^/^*^–^ mice. These data suggest that K_*c*__*a*_3.1 may also be a pharmacological target for ischemic stroke (Chen et al., 2016).

Proliferation and activation of microglia is a prominent feature in Alzheimer’s Disease (AD) (Hansen et al., 2018). In a hippocampal slice preparation, application of oligomers of Aβ (AβO) increased the expression and activity of K_*C*__*a*_3.1; in a mouse model of AD (5×FAD) and in AD patients the increase in expression and activity of K_*C*__*a*_3.1 was also observed. In both the slice preparation as well as in the mouse AD model, the application of senicapoc, an inhibitor of K_*C*__*a*_3.1, prevented proinflammatory and hLTP-impairing activities of AβO and neuroinflammation, amyloid load, and improved synaptic plasticity (Jin et al., 2019).

## Discussion

### Controversies

Depletion of microglia can result in divergent outcomes depending on the experimental model (*vide supra*). It will be important to determine whether these different outcomes are on the bases of experimental design (perturbants’ cell-specificity, dose, and timing with respect to microglial depletion/repletion kinetics) neuroanatomical lesion or other factors that affect the state of microglial polarization (*vide infra*).”

Because activated microglia can be both neurotoxic and neuroprotective, many investigators consider these diametrically opposite effects to be due to polarization between M1 (neurotoxic) and M2 (neuroprotective) phenotypes (Hanisch and Kettenmann, 2007; Ransohoff, 2016; Schoevers et al., 2016; Tang and Le, 2016; Lin et al., 2017; Zhang et al., 2017; Zabala et al., 2018; Butturini et al., 2019; Verdonk et al., 2019; Wang H. et al., 2020; Zhao et al., 2020) along the same lines as had previously been assigned to other tissue macrophages (Michelucci et al., 2009). However, this characterization has been hotly disputed (Ransohoff, 2016) and individual macrophage RNA seq data suggest a lack of exclusivity of the polarization states following traumatic brain damage (Kim et al., 2016). Therefore, this also brings into question whether modulators that reputedly transform microglia from one phenotype into another are realistic therapeutic opportunities (Song and Suk, 2017).

The concept that the CNS is an “immunoprivileged” compartment that is neither affected by, nor responsive to, peripheral inflammation has now been comprehensively debunked (Engelhardt et al., 2017). Rather, the CNS can almost be considered another lymphoid organ (Negi and Das, 2018) as well as a means whereby peripheral inflammation can be resolved (Caravaca et al., 2019).

### Fundamental Concepts and Potential Developments in the Field

Microglia migrate to the neural tube from the yolk sac during embryogenesis before the establishment of the BBB. However, microglia themselves may be dynamically involved in barrier function (Ronaldson and Davis, 2020) although a neat characterization into leak-inducing M1 and barrier-enhancing M2 phenotypes is probably invalid. Even in states in which the BBB has been disrupted, proliferation of microglia originates from precursor cells within the CNS rather than migration from outside of the CNS (Gu et al., 2016).

### Current Research Gaps

Whereas microglia require enhanced potassium ion translocation to become activated (Nguyen et al., 2017) it is not certain whether the species of ion channels involved (namely Kv1.3, K_*C*__*a*_3.1, and K_*i*__*r*_2.1) are exclusively confined to this cell-type or whether these channels exist on other types of macrophages or even non-immune cells. Further characterization of these ion channel species is required before it is definitively known whether blockade with specific inhibitors is likely to result in any off-target effect.

Studies have confirmed that attenuation of the trophic signaling of CSF-1 will result in depletion of microglia (Patel and Player, 2009; Elmore et al., 2014; Dagher et al., 2015; Rice et al., 2015; Li et al., 2017; Henry et al., 2020; Oh et al., 2020). While beneficial effects of microglial depletion have resulted in an improved outcome, for example in models of PND (Feng et al., 2017b), it is not certain whether these initial benefits may be at the expense of adverse outcomes from the loss of the protective effects of microglia (Bilbo and Schwarz, 2009; Wheeler et al., 2018; Yang et al., 2018). Furthermore, before microglial depletion can be a therapeutic strategy, a more comprehensive understanding of the time-course of microglial changes in the various injury states is needed.

### Potential Developments in the Field

Now that studies have revealed that the acute form of PND can be mitigated by depleting (Feng et al., 2017b) or preventing the activation of microglia (Ieng et al., 2020), it will be important to discover the effect of these types of perturbations on the more chronic forms of PND such as Delayed Neurocognitive Recovery (within 30 days of surgery) and Postoperative Neurocognitive Disorders (within 12 months) (Evered et al., 2018). Furthermore, therapeutic options will be enhanced by the development of microglial perturbants that have a more rapid pharmacokinetics which can then be effective, not only pre-emptively, but also after the onset of a microglial-mediated injury.

As patients with pre-existing cognitive impairment are more likely to develop postoperative delirium (Styra et al., 2019), it will be important to determine whether microglia in neurodegenerative disorders are amenable to peri-operative manipulations that prevent its activation and the onset of postoperative delirium.

The molecular mechanisms whereby activated microglia produce postoperative cognitive decline are not known in detail. It is clear that microglia are crucial for synaptic pruning during development and that there are “eat me” molecular signals needed for physiologic forgetfulness (*vide supra*); however, only recently has attention focused on the ability of microglia to engage in synaptic pruning to produce the plasticity changes of different disease states (Geloso and D’Ambrosi, 2021).

The precise state of microglial function (activated or not; phenotypic type), will be an important guide to defining treatment options. At this juncture, the only non-invasive method of defining the activation state of microglia is by imaging studies with a PET ligand that binds to the translocator protein (TSPO) that is more highly expressed on activated microglia (Forsberg et al., 2017); such an investigation requires serial scans to demonstrate a change in the individual patient. What is required is a more versatile biomarker whose activity can define activation as a binary state.

## Conclusion

The centrality of microglia for *both* protection and injury to the central nervous system (CNS) remains enigmatic. Perhaps the over-arching reason for these seemingly contradictory outcomes depends on the transformation of microglia that extends beyond a dichotomous neurotoxic (M1) and neuroprotective (M2) phenotypes. Because of its both beneficial and harmful properties, microglia may prove to be an important mediator of both CNS health and disease including Perioperative Neurocognitive Disorders.

## Author Contributions

SS: data collection and writing up draft of the manuscript. VK: data collection. JV: data collection. MM: data collection and writing up draft of the manuscript. All authors have read and approved the manuscript.

## Conflict of Interest

The authors declare that the research was conducted in the absence of any commercial or financial relationships that could be construed as a potential conflict of interest.
